# Selection and Evaluation of *Staphylococcus xylosus* as a Biocontrol Agent against Toxigenic Moulds in a Dry-Cured Ham Model System

**DOI:** 10.3390/microorganisms8060793

**Published:** 2020-05-26

**Authors:** Eva Cebrián, Félix Núñez, Fernando J. Gálvez, Josué Delgado, Elena Bermúdez, Mar Rodríguez

**Affiliations:** Food Hygiene and Safety, Meat and Meat Products Research Institute, Faculty of Veterinary Science, University of Extremadura, 10003 Cáceres, Spain; evcebrianc@unex.es (E.C.); fnunez@unex.es (F.N.); fegalvezs@alumnos.unex.es (F.J.G.); jdperon@unex.es (J.D.); bermudez@unex.es (E.B.)

**Keywords:** biocontrol, *S. xylosus*, toxigenic mould, ochratoxin A, aflatoxins, cyclopiazonic acid, dry-cured ham

## Abstract

Toxigenic moulds can develop on the surface of dry-cured meat products during ripening due to their ecological conditions, which constitutes a risk for consumers. A promising strategy to control this hazard is the use of antifungal microorganisms usually found in these foods. However, to date, the effectiveness of gram-positive catalase-positive cocci (GCC+) has not been explored. The aim of this work was to select GCC+ isolates with antifungal activity to study its effectiveness in a dry-cured ham model system at the environmental conditions reached during the ripening. Forty-five strains of GCC+ were evaluated and the isolate *Staphylococcus xylosus* Sx8 was selected to assess its efficacy at two different concentrations (10^6^ and 10^4^ cfu/mL) against *Penicillium nordicum*, *Aspergillus flavus*, *Aspergillus parasiticus,* and *Penicillium griseofulvum* at 15, 20, and 25 °C. The results showed that the inoculation of 10^6^ cfu/mL of *S. xylosus* completely inhibited the growth of most fungi. In addition, in the presence of this strain at 10^4^ cfu/mL, a significant reduction in fungal growth and mycotoxins production was observed at the three temperatures studied. In conclusion, *S. xylosus* Sx8 possesses great potential as a biological agent to control toxigenic moulds in dry-cured meat products.

## 1. Introduction

Moulds become the predominant population on the surface of dry-cured meat products due to their ability to adapt to the environmental conditions reached during their ripening [1,2,3]. These microorganisms significantly contribute to the development of the characteristic sensorial attributes of these foods [4,5]. However, it is well known that moulds isolated from this type of products are capable of producing mycotoxins [2,3,6].

Ochratoxin A (OTA) is the most prevalent mycotoxin in meat products [7]. It can be produced by several moulds species such as *Aspergillus westerdijkiae*, *Penicillium verrucosum,* and *Penicillium nordicum* [8,9,10], although *P. nordicum* is the most common ochratoxigenic mould isolated from dry-cured meats [8,11]. OTA is nephrotoxic, being involved in kidney diseases and urinary tract tumors [12] and it has been classified by the International Agency for Research on Cancer (IARC) as a possible human carcinogen in the 2B category [13]. Moreover, the presence of other mycotoxins in dry-cured ham such as aflatoxins (AFs) [14,15,16,17] and cyclopiazonic acid (CPA) [18] has also been reported.

AFs are mainly produced by *Aspergillus flavus* and *Aspergillus parasiticus* and they can be found in dry-cured meat products [15,16,17,19,20]. They are immunosuppressive and hepatotoxic and have been classified by the IARC as carcinogenic to humans in the 1A group [21]. On the other hand, CPA has been detected in dry-cured ham [18,22] and can be produced by species frequently isolated in this product such as *Penicillium griseofulvum* [22,23]. CPA provokes weight loss, nausea, muscle necrosis, convulsions and neurochemical and mutagenic toxicity due to a specific inhibitor of calcium-dependent ATPase in the sarcoplasmic reticulum [23].

Therefore, it is of utmost importance to control the growth of toxigenic moulds and their mycotoxin production. Among the different antifungal strategies, one of the most promising for dry-cured meat products is the use of microorganisms usually found in them as bioprotective agents. In this sense, several yeasts [12,24,25,26,27,28], non-toxigenic moulds [25,29,30,31], and lactic acid bacteria [32,33] have shown their capacity to reduce the development of toxigenic moulds and decrease mycotoxins production and they have been proposed as potential protective cultures in dry-cured meat products. However, the possibility of using gram-positive, catalase-positive cocci (GCC+) in dry-cured ham has not been evaluated yet.

GCC+, including coagulase-negative *Staphylococcus* sp., *Micrococcus* sp. and *Kocuria* sp., are one of the predominant microbial groups for most of the ripening time in dry-cured ham [34]. Among them, the most prevalent are several species of coagulase-negative staphylococci such as *Staphylococcus equorum*, *Staphylococcus saprophyticus* and *Staphylococcus succinus*, being the predominant species *Staphylococcus xylosus* [35,36,37]. These microorganisms provide beneficial effects such as colour stabilization because of its ability to reduce nitrates to nitrites and thus, to form nitrosylmyoglobin; inhibition of rancidity due to its catalase activity that breaks down hydrogen peroxide; and flavour development as a result of proteolytic and lipolytic activities [34,38,39,40]. For all these reasons, *Staphylococcus* sp. is usually used as a starter culture in dry-cured meat products manufacturing [38,39], being frequently added at levels around 5–6 log cfu/g and reaching counts higher than 7–8 log cfu/g during the processing [41]. Therefore, it can be of great interest in the evaluation of the antifungal potential of GCC+ as a possible protective culture to control mycotoxins production in dry-cured meat products.

However, some GCC+, including coagulase-negative staphylococci isolated from dry-cured meat products, are able to produce staphylococcal enterotoxins [34]. Then, before a staphylococcus isolate was proposed as a biocontrol agent, it must be verified that it is not an enterotoxin-producing strain.

The main objective of this work was the evaluation of the antifungal potential of non-enterotoxigenic GCC+ isolated from dry-cured ham against toxigenic moulds to select a candidate to be proposed as a protective culture. The effectiveness of the selected isolate was studied in a dry-cured ham-based agar at environmental conditions usually found during the ripening of dry-cured meat products.

## 2. Materials and Methods

### 2.1. Bacteria and Fungi

A total of 45 GCC+ and 8 mycotoxin-producing moulds were used in this study. The GCC+ strains were isolated from the surface of dry-cured Iberian hams during their processing using mannitol salt agar (MSA, Conda Pronadisa, Madrid, Spain) as described by Rodríguez et al. [37]. The mycotoxin-producing moulds were obtained from different culture collections: *P. nordicum* FHSCC 15 (Pn15) from the Food Hygiene and Safety Culture Collection at the University of Extremadura (Cáceres, Spain); *P. nordicum* CBS 323.92 (Pn92), and *A. flavus* CBS 573.65 (Af65) from the Centraalbureau voor Schimmelcultures (Utrecht, Netherland); *P. nordicum* BFE 856 (Pn856) from the Federal Research Centre for Nutrition and Food (Kulmbach, Germany); *A. parasiticus* CECT 2681 (Ap2681) and *P. griseofulvum* CECT 2919 (Pg2919) from the Spanish Type Culture Collection (Valencia, Spain); *P. griseofulvum* IBT 14319 (Pg14319) from the Type Culture Collection of the Department of Biotechnology from the Technical University of Denmark, and *A. westerdijkiae* 6B (Aw6B) was kindly supplied by Dr. Paula Rodrigues from the Mountain Research Centre, Polytechnic Institute of Bragança (Portugal).

### 2.2. Staphylococcal Enterotoxin Analysis

A TECRA™ Staphylococcal Enterotoxin Visual Immunoassay (TECRA Diagnostics, Roseville, Australia) for staphylococcal enterotoxins types A to E was used. All the 45 GCC+ isolates were grown in brain heart infusion (BHI, Conda Pronadisa) at 37 °C for 18 h. After centrifugation at 10,000× *g* for 20 min, the supernatants were collected and tested according to the manufacturer’s instructions.

### 2.3. 16S rDNA Sequence Analysis

The isolates with the highest antifungal activity were identified by 16S rDNA sequencing. Total genomic DNA was extracted from 1 mL overnight cultures in BHI broth (Conda Pronadisa), using a Masterpure complete DNA and RNA Purification Kit (Lucigen, Middleton, WI, USA), and processed according to the manufacturer’s instructions. The quantity and quality of DNA were spectrophotometrically determined in a biophotometer (Eppendorf AG, Hamburg, Germany). Universal primers 27F (5′-AGAGTTTGATCCTGGCTCAG-3′), and 1492R (5′-TACGGTTACCTTGTTACGACTT-3′) were used to amplify the 16S rDNA gene. The amplification program consisted of initial denaturation at 95 °C for 5 min; 30 cycles of denaturation at 94 °C for 30 s, annealing at 58 °C for 30 s, and extension at 72 °C for 40 s; and a final extension step at 72 °C for 1 min in a thermal cycler (Eppendorf AG 22331). Cycle sequencing reaction products were purified with a Sephadex column. Sequence analysis of PCR products was carried out by the Applied Bioscience Techniques Service of the University of Extremadura (STAB, Badajoz, Spain). The sequence results obtained were aligned with the NCBI database using the BLAST program (http://blast.ncbi.nlm.nih.gov, NCBI, Bethesda MD, USA). The identities of the isolates were determined on the basis of the highest score.

### 2.4. Inocula Preparation

The inocula of cocci were obtained by inoculating 100 µL of a culture preserved at −80 °C in BHI broth (Conda Pronadisa) and incubating at 30 °C for 48 h. Then, the count of microorganisms was obtained by plating them on MSA. Once the concentration of cells was known, the appropriate dilutions were made to achieve the concentrations of 10^8^, 10^6^ and 10^4^ cfu/mL used as inoculum.

The moulds were grown on potato dextrose agar (PDA, Conda Pronadisa) at 25 °C for 7 days. The spores were collected using 4 mL of saline phosphate buffer (PBS) and rubbing the surface with a glass rod. The spore suspensions were quantified by using a Thoma counting chamber Blaubrand^®^ (Brand, Germany) and adjusted to 10^6^ spores/mL and used as inoculum.

### 2.5. Effect of GCC+ on Growth of Ochratoxigenic Moulds

The antifungal activity of 45 strains of GCC+ was evaluated against *A. westerdijkiae* 6B and *P. nordicum* Pn15. Suspensions of 10^8^ cfu/mL cocci were mixed with 20 mL of melted meat extract agar (MEA; 30 g/L of meat extract (Conda Pronadisa), 20 g/L Bacto agar (Conda Pronadisa), 30 g/L of NaCl and 1000 mL of distilled water), and poured into Petri dishes, which were left for 2 h to solidify. After the solidification, drops of 10 µL at a concentration of 10^6^ spores/mL of each mould inoculum were inoculated at a single point on the centre of each plate. Drops of mould spore suspension were also placed on GCC+-free MEA plates, which were used as control. The fungal growth was estimated after 5 days of incubation at 25 °C by measuring the diameter of the mould colonies in two perpendicular directions. The experiment was carried out in triplicates.

### 2.6. Effect of S. xylosus on Growth and Mycotoxins Production by Mycotoxigenic Moulds

This study was carried out in dry-cured ham-based agar in Petri dishes of 5 cm diameter against 7 toxigenic fungi (*P. nordicum* Pn15, Pn 92 and Pn856, *A. flavus* Af65, *A. parasiticus* Ap2681, and *P. griseofulvum* Pg2919 and Pg14319). It was made by mixing 30 g/L of lyophilised dry-cured ham, 20 g/L Bacto agar (Conda Pronadisa), 50 g/L of NaCl, and 1000 mL of distilled water. Finally, the water activity (a_w_) was 0.95. For each fungus, two batches were prepared (treated and untreated control), and three temperatures were used (15, 20, and 25 °C), like those found during the dry-cured ham ripening [37]. A control batch was inoculated only with the mould and another batch was inoculated with the mould and *S. xylosus*. First, 50 µL of the solution containing bacteria (10^6^ or 10^4^ cfu/mL) were spread on the surface of the culture medium in the corresponding plates to reach an initial count of 2.5·10^3^ and 2.5·10 cfu/cm^2^, respectively. These concentrations of GCC+ are commonly found in dry-cured hams [37]. Then, 2 μL of the mould spore suspension (10^6^ spores/mL) were inoculated superficially at a single point in the plate centre in all batches. The experiment was carried out in triplicates.

#### 2.6.1. Growth Assessment of Toxigenic Moulds

The diameter of the growing colonies was measured each day in two perpendicular directions until the mould colonies covered the full surface of the plate in the control batches, during a maximum of 30 days.

#### 2.6.2. Extraction and Quantification of Mycotoxins

After removing the mycelium, 1 g of agar was picked up from the centre of fungal colonies and it was stored at −20 °C until the extraction of each mycotoxin.

##### OTA Extraction

OTA was extracted following the QuEChERS methodology proposed by Kamala et al. [42] with modifications. One gram of agar was mixed with 2 mL of water acidified with 0.1% acetic acid, and shaken for 30 s. Then, 2 mL acetonitrile acidified with 0.1% acetic acid was added and shaken for 1 min. Thereupon, 0.4 g NaCl and 1.6 g MgSO_4_ were added and shaken manually for 30 s. The samples were centrifuged for 5 min at 5000 rpm, and an aliquot of 1 mL supernatant was collected. Finally, the samples were diluted to 1:1 with water and filtered through a 0.22 μm pore-size nylon membrane (RephiLe Bioscience, Philadelphia, PA, USA).

##### Aflatoxins Extraction

For the extraction of AFs, the methodology described by Peromingo et al. [14] was applied. One gram of sample was mixed with 5 mL of chloroform and shaken at 150 rpm during 24 h in an orbital shaker. Then, the supernatant was transferred to amber tubes and dried in the dark. Reconstitution was carried out by adding 500 µL of HPLC-grade acetonitrile. Finally, the samples were diluted 1:1 with ultrapure water and filtered through a 0.22 μm pore-size nylon membrane (RephiLe Bioscience).

##### CPA Extraction

CPA was extracted following the QuEChERS procedure described by Peromingo et al. [18]. One gram of sample was mixed with 1 mL of water containing 0.1% of acetic acid, and mixed with a vortex for 30 s. Thereupon, 0.2 g NaCl and 0.8 g MgSO_4_ were added, and shaken manually for 30 s. The samples were centrifuged for 5 min at 5000 rpm, and an aliquot of 0.5 mL supernatant was collected into amber glass roller bottles. The extracts were dried in the dark and then, were reconstituted with 500 µL of HPLC-grade acetonitrile. Finally, the samples were diluted 1:1 with ultrapure water and filtered through a 0.22 μm pore-size nylon membrane (RephiLe Bioscience).

##### Recovery Experiments and Linearity Range

Recovery for OTA, AFs, and CPA extraction methods had been previously calculated [18,43] showing a recovery rate >90%. Linearity was estimated in the working range for every mycotoxin by spiking the acetonitrile recovered from non-inoculated samples extracted with QuEChERS in order to subtract the matrix effect. For OTA, the calibration curve was done using 14 calibration levels from 0.05 to 100 ng/mL. For AFB_1,_ two curves were made, the first one with 6 calibration levels from 0.01 to 1 ng/mL, and the second one included 8 calibration levels from 1 to 30 ng/mL. Similarly, two curves were prepared for AFG_1_, one with 6 calibration levels from 0.01 to 1 ng/mL, and the other one with 7 calibration levels from 1 to 25 ng/mL. Finally, for CPA 11, calibration levels were used from 1 to 1500 ng/mL. Each calibration curve showed an R^2^ ≥ 0.99 for every working range.

##### Mycotoxin Quantification by Orbitrap Q Exactive Plus Analysis

**Figure 1:**
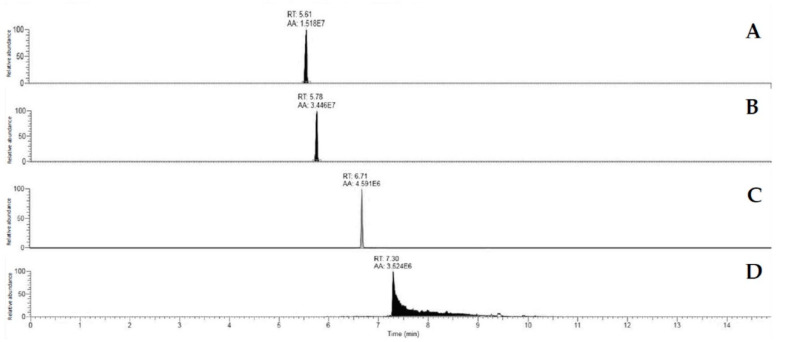
Chromatograms showing the signal obtained from Q Exactive Plus apparatus for the four mycotoxins tested (A: AFG_1_, B: AFB_1_, C: OTA and D: CPA).

The chromatographic analysis was common for every mycotoxin determination. Ten microliters of every sample were injected in a Dionex Ultimate 3000 UHPLC apparatus (Thermo Fisher Scientific, Waltham, MA, USA) equipped with a degassing system, a Quaternary HPLC pump, an autosampler device, and a thermostated (45 °C) Thermo Fisher Accucore Aq C18 (150 × 2.1 mm) column 2.6 μm particle size. Mobile phases were prepared with HPLC grade solvents: phase A (H_2_O 0.1% formic acid (FA)), phase B (acetonitrile 0.1% FA). The flow was 0.3 mL/min, and the mycotoxin separation was performed with a pre-conditioning of 10% B for 3 min, separation slope for 5 min, 25–98% B, column washing (98% B) for 6 min and 1 min of postconditioning (10% B). AFG_1_ eluted by 5.61 ± 0.1 min, and AFB_1_ by 5.78 ± 0.1 min, showing successful discrimination among them (Figure 1). OTA was eluted by 6.71 ± 0.1 min, and CPA by 7.30 ± 0.1 min.

The identification and quantification of mycotoxins was carried out by using a Q Exactive Plus mass spectrometer (Thermo Fisher Scientific). For AFs determination, the Full Scan methodology was utilised. OTA and CPA were quantified by using T-SIM mode, setting an isolation window of 2 *m/z*.

For every analysis, an ESI source (HESI II, Thermo Fischer Scientific) operating in positive ion mode (ESI+) was used. Ion source parameters in this positive mode were spray voltage 4 kV, sheath gas (N_2_ *>* 95%) 30, auxiliary gas (N_2_ *>* 95%) 10, capillary temperature 350 °C, S-lens RF level 55, auxiliary gas heater temperature 350 °C.

For t-SIM analysis, the value for automatic gain control (AGC) target was set at 5 × 10^4^; for Full Scan, it was 1 × 10^6^, and a scan rate in the range between 150 and 800 *m*/*z*. Every analysis was carried out with a resolution of 70,000 full widths at half maximum (FWHM). Data analysis and processing were performed using the FreeStyle™ software, v. 1.5 (Thermo Fisher Scientific). Each mycotoxin was identified by its retention time and its exact mass at ≤5 ppm.

##### Mycotoxin Determination Sensitivity

Limit of detection (LOD) and limit of quantification (LOQ) were calculated by using the same acetonitrile matrix from untreated samples undergone to QuEChERS method, and spiked with OTA, AFs or CPA standards, as the lowest evaluable concentration level at which the qualifier ion signal exceeds the noise level by a factor of 3.5 and 10, respectively. Being the LOD and LOQ 0.005 ng/mL and 0.01 ng/mL for AFB_1_, 0.036 ng/mL and 0.05 ng/mL for AFG_1_, 0.014 ng/mL and 0.05 ng/mL for OTA, and 0.29 ng/mL and 1 ng/mL for CPA.

### 2.7. Statistical Analysis

Statistical analysis was performed using the IBM SPSS Statistics 22.0 software (IBM, Armonk, NY, USA). All data were tested for normality using the Shapiro-Wilk test, and the homogeneity of variances using the Levene test. All data sets failed the normality test, so variable transformation was performed to improve normality or homogenise the variances but without any success. The analysis of non-parametric data was performed using the Kruskal-Wallis test. After that, the Mann-Whitney U test was applied to compare the mean values obtained. The statistical significance was set at *p* ≤ 0.05.

## 3. Results

### 3.1. Enterotoxigenic Potential of Gram-Positive, Catalase-Positive Cocci

The enterotoxigenic potential of the 45 GCC+ isolated from dry-cured hams was evaluated by the TECRA^TM^ immunological assay. The obtained results revealed that none of these isolates produced detectable amounts of staphylococcal enterotoxins.

### 3.2. Selection and Identification of Gram-Positive, Catalase-Positive Cocci Isolates with Antifungal Activity

**Figure 2:**
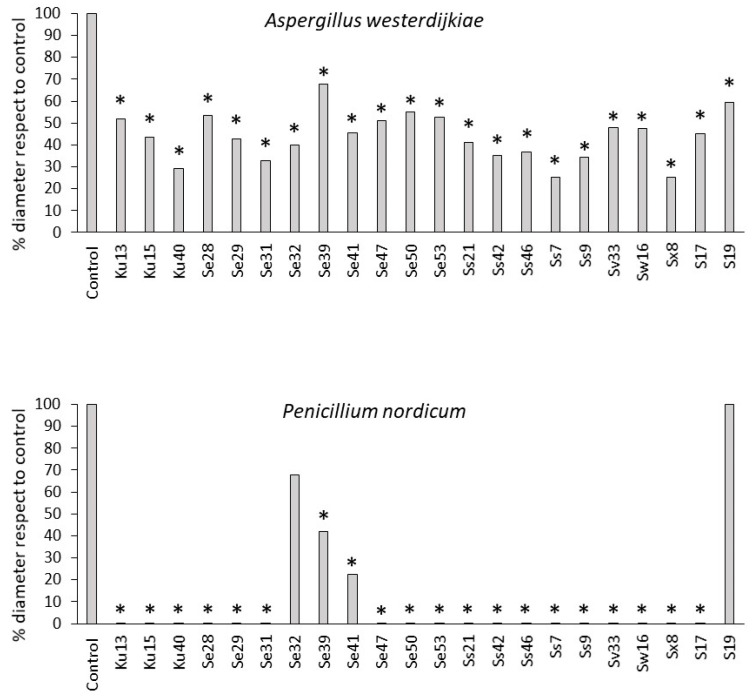
Antifungal effect of Gram + catalase + cocci on *A. westerdijkiae* and *P. nordicum* growth represented as the percentage of the diameter of the mould colonies in the presence of cocci with respect to the growth of the control mould in the absence of the bacteria. * Significant differences (*p* ≤ 0.05) for treated samples with respect to control are indicated by an asterisk.

The antifungal activity of 45 GCC+ isolated from dry-cured ham at different stages of ripening was studied. The screening revealed that 22 isolates (48.9%) produced a significative decrease in the growth of at least one of the reference moulds used (Figure 2). These 22 active isolates were then identified by sequencing of 16S rDNA region, resulting in 3 *Kocuria uropygioeca*, 9 *S. equorum*, 5 *S. saprophyticus*, 1 *Staphylococcus vitulinus*, 1 *Staphylococcus warneri*, 1 *S. xylosus*, and 2 isolates of uncertain identification between *Staphylococcus pasteuri* and *S. warneri* (Table 1).

In general, *P. nordicum* was more sensitive than *A. westerdijkiae*, since it did not grow in the presence of 18 tested GCC+. Although *A. westerdijkiae* was able to grow in all cases, all 22 active GCC+ reduced their development, recording its greatest inhibition in the presence of *S. saprophyticus* Ss7 and *S. xylosus* Sx8. Then, *S. xylosus* Sx8 was selected to conduct the following experiment according to its overall activity against both ochratoxigenic moulds, and considering that this species is predominant during the processing of dry-cured ham.

### 3.3. Antifungal Activity of S. xylosus in a Dry-Cured Ham Model System

#### 3.3.1. Effect on Mould Growth

**Figure 3:**
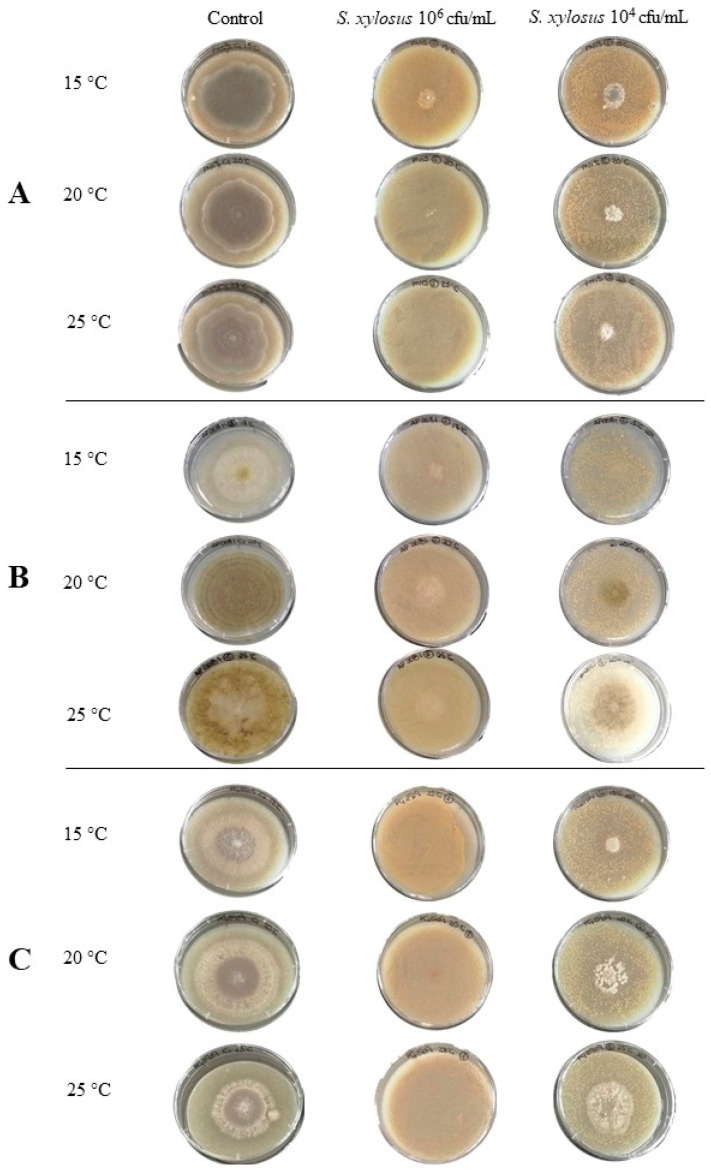
Effect of *S. xylosus* Sx8 at different initial concentrations (10^6^ and 10^4^ cfu/mL) on the final growth of *P. nordicum* Pn15 (**A**), *A. parasiticus* Ap2681 (**B**), and *P. griseofulvum* Pg2919 (**C**) at 15, 20, and 25 °C.

**Figure 4:**
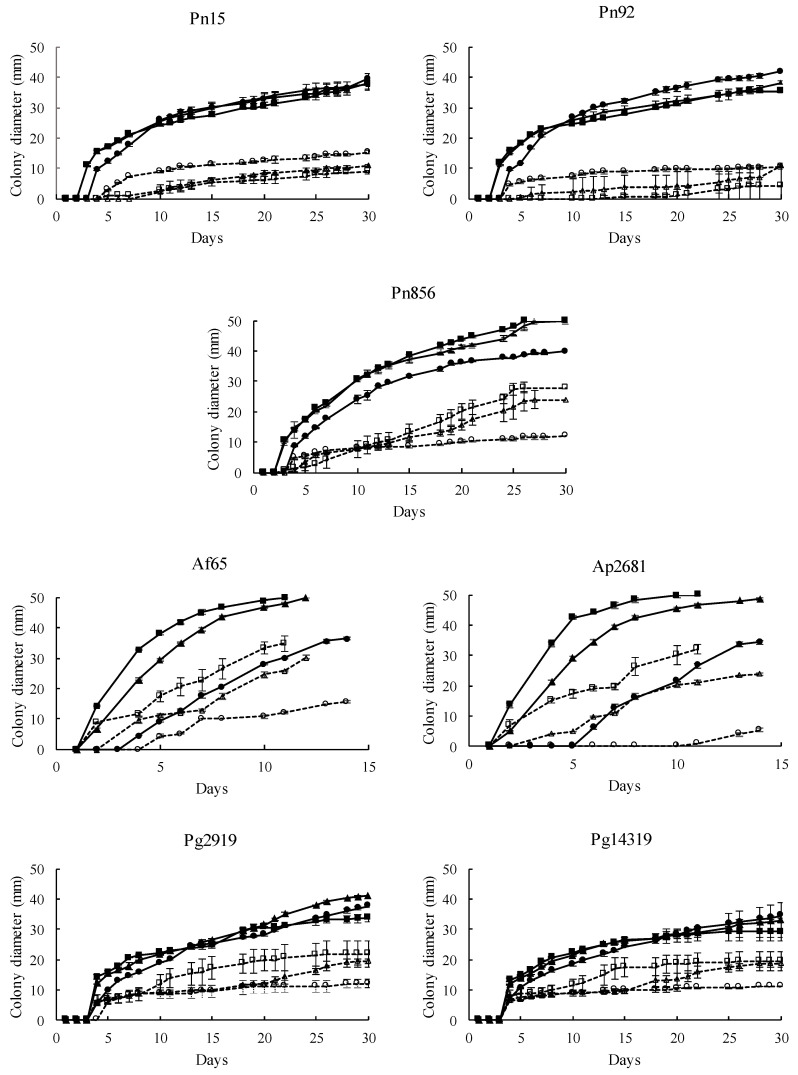
Growth of OTA-producing *P. nordicum* (Pn15, Pn92, Pn856), AFs-producing *A. flavus* Af65 and *A. parasiticus* Ap2681, and CPA-producing *P. griseofulvum* (Pg2919, Pg14319) strains at 15 (●), 20 (▲), and 25 °C (■), in the presence (discontinuous line, open symbols) or absence (continuous line, closed symbols) of *S. xylosus* Sx8.

The effect of *S. xylosus* Sx8 on growth rates of *P. nordicum*, *A. flavus*, *A. parasiticus*, and *P. griseofulvum* was studied in a dry-cured ham-based medium under ecological conditions usually found during the processing of this product. All the fungal strains tested showed a satisfactory growth in this culture medium when they were individually cultured without the presence of *S. xylosus* Sx8 (untreated control batch, Figure 3). The pattern of development for the strains that produce the same mycotoxin was quite similar (Figure 4), showing *A. flavus* and *A. parasiticus* the fastest growth, as they covered the whole surface plate in a range of 11–14 days, depending on the incubation temperature. In addition, each mould reached almost the same growth levels regardless of the incubation temperature, except *A. flavus* and *A. parasiticus* whose growth was lower at 15 °C.

*S. xylosus* had a marked influence on the development of all toxigenic moulds. When the inoculum level of 10^6^ cfu/mL of Sx8 was used (reaching an initial load of approximately 2.5·10^3^ cfu/cm^2^), no growth was observed at 15–25 °C for most of the treated fungi, while in untreated control batches, moulds had covered almost the whole surface of the plates (Figure 3). When the assay was performed using 10^4^ cfu/mL of Sx8 (reaching approximately 2.5·10 cfu/cm^2^), a significant growth diminution of all *P. nordicum*, *A. flavus*, *A. parasiticus*, and *P. griseofulvum* strains was recorded at every temperature (Figure 4).

#### 3.3.2. Effect on Mycotoxin Production

The OTA amount produced by *P. nordicum* Pn15 and Pn92 in the presence of *S. xylosus* Sx8 was significantly reduced to below LOQ and LOD respectively at all tested temperatures (Table 2). For *P. nordicum* Pn856, there was a reduction in OTA production of 86.59% and 75.35% at 15 and 20 °C, respectively. However, at 25 °C, an increase of OTA production by Pn856 in the presence of the Sx8 was observed.

The production of AFB_1_ by *A. flavus* Af65 and *A. parasiticus* Ap2681 was significantly reduced in the presence of *S. xylosus* Sx8 (Table 3). In *A. flavus,* the greater reduction (99.32%) was obtained at 20 °C. The production of this mycotoxin by *A. parasiticus* Ap2681 was considerably lower in every condition, and Sx8 led to a reduction to non-detectable level at 25 °C. On the other hand, the production of AFG_1_ was also significantly reduced to non-detectable level when *A. parasiticus* was co-inoculated with *S. xylosus* Sx8 at three temperatures (Table 4).

CPA produced by *P. griseofulvum* was the mycotoxin that reached the higher concentrations in the dry-cured ham-based medium (Table 5). Sx8 triggered a significant reduction of CPA production by Pg2919 at 20 and 25 °C, and by Pg14319 at 15 and 25 °C.

## 4. Discussion

The use of lactic acid bacteria [32], yeasts [12,24,25,26,27,28,44], and non-toxigenic moulds [25,30,31,45] isolated from dry-cured meat products as protective cultures against toxigenic moulds in these foods has been widely proposed. However, to the best of our knowledge, the feasibility of using GCC+ for this purpose has not been explored yet, even though this group is among the prevailing ones in dry-cured meat products [35,36,37]. Therefore, the antifungal activity of cocci isolated throughout the processing of dry-cured ham deserved to be evaluated.

The selection of the suitable isolates to be used as protective cultures should be based not only on their antifungal activity but also on the safety criteria. In this sense, some GCC+ such as coagulase-negative staphylococci, coming from dry-cured meat products may produce staphylococcal enterotoxins [34]. Thus, the toxin-producing staphylococci must be excluded. Then, the enterotoxigenic potential of the isolates was checked by TECRA^TM^ immunological assay. None of the 45 tested cocci produced staphylococcal enterotoxins at detectable levels. Therefore, they should be considered as safe, and they could be eventually proposed as biocontrol agents.

A high ratio of active antifungal isolates was obtained, since 22 out of the 45 tested GCC+ (48.9%) showed inhibitory activity against at least one of the ochratoxigenic reference moulds used (Figure 2). The environmental conditions reached in the processing of dry-cured meat products are suitable for the persistence of GCC+ for long periods of time [37]. In this context, their antifungal ability could represent an ecological advantage in these foods in which moulds also develop extensively [2,3,22].

According to the overall results obtained against ochratoxigenic moulds, *S. xylosus* Sx8 was selected as a biocontrol agent candidate. This isolate was able to completely prevent the growth of *P. nordicum*, and was also showing the highest inhibition against *A. westerdijkiae* along with *S. saprophyticus* Ss7 (Figure 2). On the other hand, *S. xylosus* is the overwhelmingly predominant species in dry-cured meats products throughout the processing, even after 16 months of ripening [34,36]. Moreover, the *S. xylosus* strain has been proposed to be used as starter cultures for dry-cured meat products, because of its contribution to the typical flavour and coloration of these foods [46,47].

The antifungal activity of *S. xylosus* Sx8 was evaluated against a range of toxigenic moulds belonging to species commonly isolated from dry-cured meat products. Then, the screening was performed by coculturing the active Sx8 against several strains of the ochratoxigenic *P. nordicum*, the aflatoxigenic *A. flavus* and *A. parasiticus*, and *P. griseofulvum* producers of CPA in a dry-cured ham-based medium at three temperatures (15, 20, and 25 °C) usually found during the processing of this food. *S. xylosus* Sx8 decreased the growth of all the toxigenic moulds during the whole incubation period at every temperature tested. The efficiency in preventing fungal growth depended on the level of inoculation of Sx8. Then, when a rate of 10^6^ cfu/mL was inoculated (reaching about 2.5·10^3^ cfu/cm^2^ in plate surface of initial load), no mycelium development of most of the toxigenic strains studied was observed (Figure 3). Similar results were found regarding the antifungal activity of *Debaryomyces hansenii* and *Saccharomycopsis fibuligera* against *P. nordicum* and *Aspergillus ochraceus* in speck, recording a greater growth reduction with a yeasts concentration of 10^6^ cfu/mL than with 10^4^–10^2^ cfu/mL [26]. In the same way, Andrade et al. [24] found that increasing the inoculum concentration from 10^4^ to 10^6^ cfu/mL of *D. hansenii* significantly improved the growth inhibition of *P. nordicum* in culture media.

The antagonistic effect of staphylococci has been linked to the generation of antifungal volatile compounds. *S. saprophyticus* L-38 inhibited *A. flavus* conidial germination and mycelial proliferation, through the production of 3,3-dimethyl-1,2-epoxybutane [48]. The inhibition of the mycelial growth and conidial germination of the plant pathogen *Colletotrichum nymphaeae* by *Staphylococcus sciuri* MarR44 has been also attributed mainly to volatile compounds, but without ruling out the role of other metabolites such as siderophore, chitinase, protease, hydrogen cyanide, indole-3-acetic acid, and gibberellin and the biofilm formation [49]. In this sense, the production of extracellular metabolites, including volatile compounds, proteins, lytic enzymes or organic acids has been reported in a range of microorganisms with antifungal activity [44,50,51,52]. In addition, competition by nutrients and space is considered one of the most common antagonistic mechanisms of antifungal agents, including bacteria, moulds and yeasts [12,44,53,54]. Considering all these possibilities, it is necessary to conduct further researches to elucidate the mechanism of action involved in the capability of *S. xylosus* Sx8 to reduce fungal growth.

In addition to the reduction of mould growth, *S. xylosus* Sx8 significantly decreased the mycotoxin concentration in most of the conditions tested. For OTA-producing moulds, the presence of *S. xylosus* Sx8 significantly reduced (*p* ≤ 0.05) the amount of this mycotoxin regardless of temperatures. In this way, the effect of this staphylococcus strain provokes that the OTA production by *P. nordicum* Pn15 and Pn92 drops to levels below the LOQ and LOD respectively (Table 2) at 15, 20, and 25 °C. However, even though OTA production by *P. nordicum* Pn856 also decreased at 15 and 20 °C, there was an increase at 25 °C. Although supposedly a reduction in mould growth would mean a lower mycotoxin production, previous studies have shown that OTA production by *P. nordicum* and *P. verrucosum* may increase under sub-optimal growth conditions [9]. Moreover, some stressful conditions seem to stimulate secondary metabolism with the consequent increase of mycotoxin production [10,55]. In fact, fungal secondary metabolism is strongly stimulated by sub-optimal conditions like nutrient depletion or metabolites produced by other organisms [56]. Nevertheless, this phenomenon only occurs in *P. nordicum* Pn856 at 25 °C at the lowest concentration tested of *S. xylosus* Sx8, but not at any other temperatures, nor for other *P. nordicum* strains, when an outstanding reduction in OTA amount was recorded.

*S. xylosus* also triggered a decrease in the production of AFB_1_ (Table 3) by *A. flavus* Af65 and *A. parasiticus* Ap2681, in the production of AFG_1_ (Table 4) by *A. parasiticus*, and in production of CPA by *P. griseofulvum* Pg2919 and Pg14319 (Table 5) at the full range of temperature evaluated (15–25 °C).

The reduction of mycotoxins amounts by *S. xylosus* reported in the present work could be related to the lower fungal growth reported. However, other modes of action may be involved. In this sense, the ability of *S. saprophyticus* to reduce the AFB_1_ amount in cultures of *A. flavus* in corn and peanuts was linked to the abolition of mycelia growth due to the volatile 3,3-dimethyl-1,2-epoxybutane [48]. In other bacteria, such as *Bifidobacterium* and *Lactobacillus*, mycotoxin reduction is due to the enzymatic degradation or to the adsorption on the cell wall [57]. The yeasts *D. hansenii* decreased OTA and AFs production in meat substrates by the blockage in the expression of genes related to the synthesis of these mycotoxins [25,27,28]. On the other hand, the effect of *P. chrysogenum* on decreasing OTA production by *P. nordicum* has been attributed to the repression of genes of the OTA-biosynthetic pathway [25], but also to the nutritional competition that implies a reduction in the secondary metabolism [53,54]. Nevertheless, no information about the mode of action of *S. xylosus* in the mycotoxin reduction in meat substrates is still available, and further investigations are required to elucidate it.

Therefore, the use of *S. xylosus* Sx8 to control toxigenic moulds in dry-cured meat products should be considered as a promising approach, since this species is adapted to this ecological niche. However, the inoculation size of Sx8, the environmental conditions, and even the specific mould strain seem to be key factors for its effectiveness. Thus, to assure a successful outcome, a level of 2.5·10^3^ cfu/cm^2^ of this microorganism should be reached on the food surface. Its inoculation on the earlier stages of the processing will ensure its implantation in the food reaching high counts to prevent the growth of toxigenic moulds and the presence of mycotoxins. In this sense, although the counts of GCC+ decreased continuously until the end of the processing of dry-cured ham, the counts usually are higher to 10^4^ cfu/g for a long time [37].

On the other hand, according to the obtained results, the effect of *S. xylosus* Sx8 is not enough for the whole suppression of mycotoxins production by all the tested moulds, but it contributed to reducing the accumulation of these mycotoxins. This effect can be enhanced by using Sx8 in mixed cultures together with other microorganisms with complementary activities against toxigenic fungi in dry-cured meat products, such as the yeast *D. hansenii* or the mould *Penicillium chrysogenum* [25,27,28,30]. In order to design these mixed protective cultures, it is crucial to understand the mode of action of each biocontrol agent and their plausible synergistic effect. Then, further studies must be carried out to establish the antifungal mechanism of *S. xylosus* Sx8 as well as other active GCC+ isolated from dry-cured meat products to optimise the appropriate timing and managing their application during the dry-cured ham processing, in order to successfully prevent hazard posed by mycotoxins.

In conclusion, *S. xylosus* Sx8 exhibits a broad antifungal activity against important toxigenic fungi commonly found on dry-cured ham. In addition, Sx8 provokes a significant decrease on mycotoxins accumulation in meat substrate. Then, the results of the present study suggest that this staphylococcus possesses great potential as a biological agent to control toxigenic moulds in dry-cured meat products.

## Figures and Tables

**Table 1:** Identification by sequencing of 16S rDNA region of Gram + catalase + cocci with antifungal activity.

Isolate	Identification	Accession Number	% Identity
Ku13	*K. uropygioeca*	NR_157676.1	95.88
Ku15	*K. uropygioeca*	NR_157676.1	98.72
Ku40	*K. uropygioeca*	NR_157676.1	100
Se28	*S. equorum*	MK015791.1	99.86
Se29	*S. equorum*	MN229550.1	99.79
Se31	*S. equorum*	MN229551.1	99.91
Se32	*S. equorum*	MN758799.1	98.28
Se39	*S. equorum*	KY940339.1	99.85
Se41	*S. equorum*	MK796070.1	99.92
Se47	*S. equorum*	MN758799.1	99.64
Se50	*S. equorum*	KP224447.1	99.56
Se53	*S. equorum*	KY940339.1	99.86
Ss21	*S. saprophyticus*	KU922358.1	99.02
Ss42	*S. saprophyticus*	MF457583.1	99.4
Ss46	*S. saprophyticus*	MF457583.1	99.7
Ss7	*S. saprophyticus*	MN229561.1	98.52
Ss9	*S. saprophyticus*	KT717631.1	99
Sv33	*S. vitulinus*	KM378591.1	99.69
Sw16	*S. warneri*	MK414943.1	98.8
Sx8	*S. xylosus*	HM854231.1	99
S17	*S. pasteuri/S. warneri*	MH930440.1/MN181250.1	99.79
S19	*S. pasteuri/S. warneri*	MH930440.1/MN421516.1	99.79

**Table 2:** Effect of *S. xylosus* Sx8 on ochratoxin A (OTA) production (ng/g) by *P. nordicum* Pn15, Pn92, and Pn856 in dry-cured ham-based media during 30 days of incubation at 15, 20, and 25 °C.

	15 °C		20 °C		25 °C	
Mould	Control	Sx8	Control	Sx8	Control	Sx8
Pn15	3.93 ± 1.36	<LOQ *^,a^	18.76 ± 3.22	<LOQ *	66.59 ± 19.52	<LOQ *
Pn92	2.75 ± 0.49	<LOD *^,b^	26.61 ± 4.82	<LOD *	89.53 ± 14.90	<LOD *
Pn856	31.54 ± 0.07	4.23 ± 0.35 *	260.64 ± 71.93	64.26 ± 11.72 *	389.73 ± 57.65	860.13 ± 357.03 *

* Significant differences with respect to control *p* ≤ 0.05; ^a^ Values under the limit of quantification; ^b^ Values under the limit of detection.

**Table 3:** Effect of *S. xylosus* Sx8 on aflatoxin B_1_ (AFB_1_) production (ng/g) by *A. flavus* Af65, and *A. parasiticus* Ap2681 in dry-cured ham-based media during 14 days of incubation at 15, 20, and 25 °C.

	15 °C		20 °C		25 °C	
Mould	Control	Sx8	Control	Sx8	Control	Sx8
Af65	2.09 ± 0.25	0.18 ± 0.03 *	41.06 ± 6.23	0.28 ± 0.09 *	28.49 ± 3.07	2.08 ± 2.21 *
Ap2681	0.08 ± 0.02	0.01 ± 0.01 *	0.06 ± 0.05	0.01 ± 0.01	0.10 ± 0.02	<LOD *^,a^

* Significant differences with respect to control *p* ≤ 0.05; ^a^ Values under the limit of detection.

**Table 4:** Effect of *S. xylosus* Sx8 on aflatoxin G_1_ (AFG_1_) production (ng/g) by *A. parasiticus* Ap2681 in dry-cured ham-based media during 14 days of incubation at 15, 20, and 25 °C.

	15 °C		20 °C		25 °C	
Mould	Control	Sx8	Control	Sx8	Control	Sx8
Ap2681	0.73 ± 0.34	<LOD *^,a^	4.85 ± 8.24	<LOD *	0.91 ± 0.30	<LOD *

* Significant differences with respect to control *p* ≤ 0.05; ^a^ Values under the limit of detection.

**Table 5:** Effect of *S. xylosus* Sx8 on cyclopiazonic acid (CPA) production (ng/g) by *P. griseofulvum* Pg2919, and Pg14319 in dry-cured ham-based media during 30 days of incubation at 15, 20, and 25 °C.

	15 °C		20 °C		25 °C	
Mould	Control	SX8	Control	SX8	Control	SX8
Pg2919	1270.4 ± 431.9	888.5 ± 258.3	2427.8 ± 724.8	1793.9 ± 34.7 *	4492.9 ± 227.0	2694.1 ± 881.9 *
Pg14319	2804.2 ± 802.8	1160.0 ± 165.0 *	4120.4 ± 944.7	2932.9 ± 651.9	6298.7 ± 2.9	5358.9 ± 382.4 *

* Significant differences with respect to control *p* ≤ 0.05.
